# Combating Stigma Through HIV Self-Testing: New York State's HIV Home Test Giveaway Program for Sexual Minorities

**DOI:** 10.1097/PHH.0000000000001138

**Published:** 2020-02-03

**Authors:** Megan C. Johnson, Rakkoo Chung, Shu-Yin J. Leung, Zoe Edelstein, Yingchao Yuan, Susan M. Flavin

**Affiliations:** New York State Department of Health—AIDS Institute, Albany, New York (Mss Johnson and Flavin, Dr Chung, and Messrs Leung and Yuan); and New York City Department of Health and Mental Hygiene, New York, New York (Dr Edelstein).

**Keywords:** HIV, HIV self-test, human immunodeficiency virus, in-home HIV test, men who have sex with men

## Abstract

**Context:**

Gay, bisexual, and men who have sex with men (MSM) are disproportionately affected by the HIV/AIDS epidemic more than any other group. In New York State (NYS) outside of New York City (NYC), MSM accounted for 57% of new HIV/AIDS diagnoses in 2017. HIV/AIDS home testing initiatives have been effective at getting priority populations tested for HIV.

**Objectives:**

The NYS Department of Health (NYSDOH) administered the HIV Home Test Giveaway (HHTG) program through social media and mobile applications popular among the priority populations to (1) promote HIV screening among gay and bisexual men, MSM, transgender (TG), and gender queer/gender nonconforming (GNC) individuals who have sex with men, and (2) identify individuals with undiagnosed HIV infection.

**Design/Setting:**

The NYSDOH recruited participants from NYS (excluding NYC) through social media campaigns between November 2016 and January 2018. Interested individuals were directed to an online eligibility survey. Eligible participants received a coupon via e-mail for a free HIV self-test (HIVST) kit through the manufacturer's Web site. Eligible participants received a $20 to $25 Amazon online gift card if they completed a voluntary online follow-up survey regarding their test results, experiences with the HHTG, and HIV/AIDS risk behaviors. Participants were also able to request assistance from the NYSDOH with further testing and various prevention and supportive services.

**Participants:**

In total, 6190 individuals participated and 3197 (52%) were eligible and received a coupon. Of the eligible, 2022 (63%) redeemed coupons for HIVST.

**Results:**

Among eligible participants, 976 (31%) reported having never been tested for HIV. On the follow-up survey, 922 (29%) participants reported having used the HIVST kit for themselves; 203 (22%) were first-time testers; and 7 (0.8%) tested HIV reactive. Of the follow-up survey participants, 761 (59%) requested assistance with various services.

**Conclusions:**

Media campaigns were effective in promoting HIV testing among priority populations and reaching individuals who have never been tested for HIV.

## Abstract 2

Supplemental Digital Content is Available in the Text.

Gay, bisexual, and other men who have sex with men (MSM) are disproportionately affected by the HIV/AIDS epidemic in the United States more than any other group; 67% of the 40 324 new HIV/AIDS diagnoses in 2016 were attributed to male-to-male sexual contact.^1^ In New York State (NYS) outside of New York City (NYC), MSM accounted for 57.3% of new HIV/AIDS diagnoses in 2017.^2^ Moreover, it is estimated that 1 in 6 gay and bisexual men with HIV/AIDS are unaware of their serostatus.^1^ Thus, the Centers for Disease Control and Prevention (CDC) recommends frequent HIV screening for individuals engaging in HIV risk behaviors.^3^ To support screening recommendations, the NYS Department of Health (NYSDOH) has made significant efforts to routinize HIV screening in medical settings and has been funding rapid testing initiatives for priority populations including MSM since the onset of the HIV/AIDS epidemic in high prevalence communities.

HIV rapid point-of-care testing technologies have been developed to expand and facilitate the availability of the over-the-counter HIV self-test (HIVST). While there are still concerns about HIV self-testing (eg, the lack of counseling, linkage to care, and sexually transmitted infection [STI] testing),^4^ a growing body of evidence suggests that HIVST can be a tool to increase access to HIV screening and improve sexual health. For example, many MSM prefer to test for HIV in privacy rather than at clinical settings or testing sites.^5^–^9^ Also, online purchase of the HIVST kit can be an option for those who are reluctant to purchase the HIVST kit from pharmacists or cashiers.^10^,^11^ In addition, many MSM use the HIVST kit and receive the results in the presence of partners, which not only increases trust and honesty but also encourages safer sexual behaviors,^5^,^12^–^14^ and has been associated with the cessation of sexual activities with casual partners in the case of positive results.^15^,^16^ These advantages make HIVST programmatically useful to encourage HIV screening among MSM and other marginalized and stigmatized priority populations such as transgender (TG) or gender queer/gender nonconforming (GNC) people who have sex with men.

Many studies and programs have tried various ways to distribute the HIVST kit to MSM via advertisements placed on social media platforms and mobile applications (social media, hereafter),^5^,^17^–^21^ peer recruitment through social networks,^12^,^14^,^22^,^23^ community-based organization outreach,^10^,^24^ and through venue-based approaches.^25^ Comparatively, social media has proven most efficient with respect to recruitment of MSM.

Since 2015, the New York City Department of Health and Mental Hygiene (NYCDOHMH)^26^,^27^ had implemented an HIV Home Test Giveaway (HHTG) program in NYC. The NYSDOH tailored this model and launched a rest of state giveaway in all other locations of NYS outside of NYC. Since fall of 2016, the NYSDOH and the NYCDOHMH have collaboratively conducted the HHTG programs simultaneously and recruited participants through social media popular among the priority populations. While this study is based on the NYS HHTG (outside of NYC), both HHTG programs in NYS and NYC are inseparable in important respects. First, it demonstrates the merits and potential of cross-jurisdictional collaboration. The collaboration with NYC is particularly beneficial because many people migrate daily between NYC and the other counties of NYS. Second, because it is run in conjunction with the NYC program, the HHTG is the first statewide HIVST distribution initiative in the United States. The HHTG can provide a model that other states may consider adopting. Plus, the NYS HHTG has a unique feature: since 2017, the NYS HHTG participants have been given the opportunity to request additional services such as STI testing and preexposure prophylaxis (PrEP) referrals by allowing the NYSDOH staff to contact them via e-mail.

The NYS HHTG intends to (1) promote HIV screening among MSM/TG/GNC individuals who have sex with men and (2) identify individuals with undiagnosed HIV infection. This study describes the NYS HHTG (outside of NYC) and reports key findings from its evaluation.

## Methods

**FIGURE 1:**
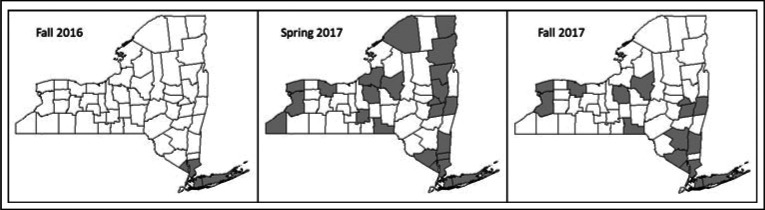
Target Counties of Media Campaigns of the NYS HHTG, Rounds 1, 2, and 3^a^ Abbreviations: HHTG, HIV Home Test Giveaway; NYC, New York City; NYS, New York State. ^a^All NYS residents (outside of NYC) were eligible, while media campaigns were targeted to the shaded counties.

**FIGURE 2:**
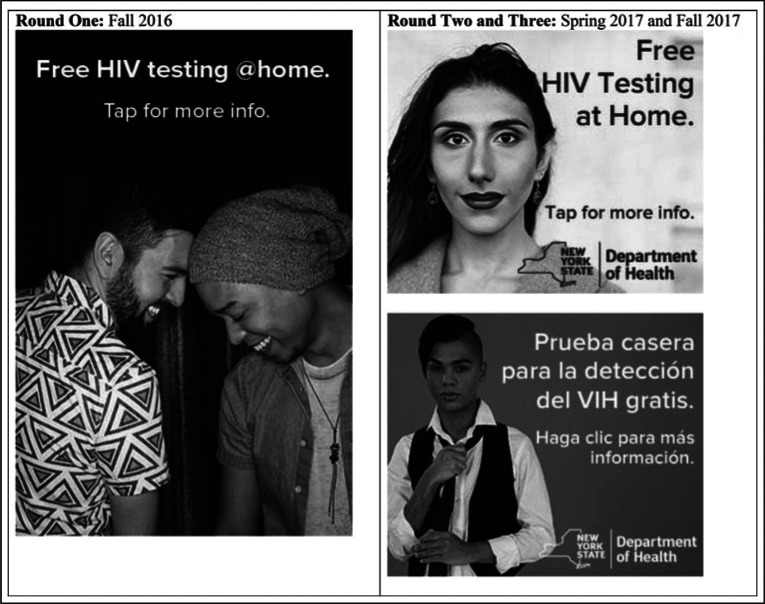
Sample Social Media Messaging^a^ ^a^Reprinted with permission from the New York State Department of Health.

In collaboration with the NYCDOHMH and the manufacturer of the HIVST kit, the NYSDOH implemented the HHTG throughout NYS excluding NYC while the NYCDOHMH implemented the HHTG within NYC, collectively serving all NYS residents by one of the 2 HHTG programs. Participants were recruited through media campaign advertisements on popular social media and social networks (such as Grindr, Facebook, Twitter, Instagram, Jack'd, Scruff, Black Gay Chat, Hornet). For Facebook, Twitter, and Instagram, our campaigns were targeted to MSM/TG/GNC who had interest in sexual contact with men. In addition, for Twitter, specific MSM/TG/GNC-friendly handles were applied to tweets to increase click-through rates to the eligibility survey. There were 3 rounds of the HHTG media campaigns: (1) November-December 2016, (2) May-June 2017, and (3) November 2017-January 2018. Three media campaigns resulted in 24 957 223 ad-impressions and 155 506 click-throughs to the online eligibility survey. While the study design (ie, the research portion of the NYS HHTG program, such as the methods of recruitment and data collection) did not change throughout the 3 rounds of the NYS HHTG, the geographical targets of media campaigns changed depending on the programmatic considerations and budget availability. Each round of the media campaign was strategically targeted to high needs areas of NYS (see Figure 1) with increased/increasing HIV infection and other signs of elevated risk for HIV infection in the communities.^28^ For the initial round in 2016, which followed the success of the NYCDOHMH's 2015 pilot, the NYSDOH established a media campaign geofence (ie, catchment area) in regions of NYS contiguous with NYC (ie, Long Island and Westchester and Rockland counties). The campaign advertisements and images depicted gay, bisexual, and other MSM and were exclusively in English (see Figure 2). Based on the success of round 1, the NYSDOH expanded the HHTG geofence in round 2 (spring 2017) to all regions of NYS (excluding NYC) by prioritizing zip codes with increased trends of HIV prevalence, increased number of new HIV/AIDS diagnoses, increased number of reported naloxone administrations by law enforcement, and recommendations from regional HIV advisory body members. To further prioritize the geofence in round 3 (fall 2017), in addition to the criteria used to establish the geofence in round 2, the NYSDOH prioritized zip codes by rate of increase in the number of new HIV/AIDS diagnoses over a 5-year period and relied on real-time new diagnosis data to shift geofences as needed. Rounds 2 and 3 contained campaign advertisements and images used in round 1 and expanded to include images of TG and GNC people and messaging in both English and Spanish (see Figure 2).

Individuals who clicked the HHTG advertisements were redirected to an eligibility survey for a free HIVST kit. Eligible participants had to be (1) a male or TG/GNC person who had had sex with a man in the past 12 months, (2) at least 18 years old, (3) residing in NYS outside of NYC, and (4) never diagnosed with HIV/AIDS. All participants, regardless of their eligibility, were provided with information on HIV/AIDS prevention and treatment, including available resources and support services. All eligible participants were asked for an e-mail address to receive a coupon that they could redeem for a rapid HIVST kit (OraQuick In-Home HIV Test) delivered to their home address at no cost.

Four to 8 weeks after the completion of the eligibility survey, all eligible participants were invited to a voluntary online follow-up survey regarding (1) their experiences with HIVST, (2) their behaviors that put them at risk of HIV infection such as condomless sex, and (3) HIV/AIDS prevention measures such as condom use, PrEP and postexposure prophylaxis (PEP), as well as (4) their HIVST results for those who tested. Unlike other home test giveaways, the follow-up survey provided all participants with the option to request assistance from the NYSDOH. In round 1, participants reporting a positive result were offered assistance with linkage to HIV/AIDS medical care, partner notification, and other prevention and supportive services. In rounds 2 and 3, assistance was expanded to all participants regardless of their self-reported HIV/AIDS status to include assistance with further testing for HIV, PrEP referrals, linkage to HIV/AIDS medical care, partner notification, and other prevention and supportive services. The follow-up survey was active for 8 to 12 weeks after each media campaign. As an incentive, an Amazon online gift card for $20 to $25 was e-mailed to every participant who had completed the follow-up survey. All participants were given an option to choose the preferred language (either English or Spanish) for both the eligibility and follow-up surveys. Both surveys were hosted on Health Insurance Portability and Accountability Act (HIPAA)–compliant platforms. No identifiable information was collected in the follow-up survey. E-mail addresses were the only identifiable information collected in the eligibility survey for the purpose of distributing the coupons to eligible participants. E-mail addresses were kept separate from the other survey data in password-protected files and stored on a secure server location only accessible by study personnel.

We used descriptive analysis (eg, frequency tables and cross-tabulations) of the participant data from the eligibility survey and the follow-up survey to examine the characteristics of participants by last HIV test, self-reported test result, and participant follow-up request. The NYS HHTG was approved by the NYSDOH institutional review board (IRB) on 26 August 2016 (project ID 941637-2; reference # 16-043) and renewed on August 15, 2017.

## Results

### Sample characteristics

Table 1 shows the sample characteristics of persons who completed the eligibility survey. Counts of unique participants (by e-mail) are displayed by round and in total, and cascade into smaller numbers by their eligibility, follow-up survey completion, and the HHTG experiences. Of 6190 interested participants who completed the eligibility survey, 3197 (51.6%) were eligible. Of the eligible participants, 2022 (63.2%) redeemed the coupon for an HIVST kit and 1510 (47.2%) completed the follow-up survey. Of the 1114 participants who redeemed the coupon and completed the follow-up survey, 922 (82.8%) reported using the HIVST kit to test themselves and 98.1% reported that they would be likely to recommend the HHTG to a friend.

**TABLE 1:** Sample Characteristics and HIV Test Results by Round for NYS HHTG

	Round^a^			Total
	1	2	3	
*Completed the eligibility survey*	503	3709	1978	6190 (100.0%)
*Eligible participants*	405	1791	1001	3197 (51.6%)
Redeemed coupon for test kit	267	1184	571	2022 (63.2%)
Did not redeem coupon	138	607	430	1175 (36.8%)
*Ineligible participants*	98	1918	977	2993 (48.4%)
Eligible, but no e-mail	5	182	67	254 (8.5%)
Eligible, but outside NYS^b^	N/A^c^	885	597	1482 (49.5%)
Ineligible	93	851	313	1257 (42.0%)
*Completed the follow-up survey* d	211	744	555	1510 (47.2%)
*Redeemed and completed the follow-up survey*	180	613	321	1114 (34.8%)
Have not used the test	25	76	36	137 (12.3%)
Tested someone else	6	25	11	42 (3.8%)
Tested themselves	146	505	271	922 (82.8%)
HIV negative	141	492	259	892 (96.7%)
Cannot understand	0	0	1	1 (0.1%)
Indeterminate	0	1	4	5 (0.5%)
No answer	4	10	3	17 (1.8%)
HIV reactive	1	2	4	7 (0.8%)
Confirmed HIV positive	0	1	4	5 (71.4%)
Confirmed HIV negative	0	0	0	0 (0.0%)
Waiting for confirmatory test results	0	1	0	1 (14.3%)
No answer	1	0	0	1 (14.3%)

Abbreviations: HHTG, HIV Home Test Giveaway; NYC, New York City; NYS, New York State.

^a^Round 1 = fall 2016; round 2 = spring 2017; round 3 = fall 2017.

^b^Participants who reported their residence zip codes outside of NYS; NYC residents participated in the NYC HHTG and thus were excluded from this article.

^c^No data available for participants from outside of NYS.

^d^All eligible participants who completed the follow-up survey, regardless of their redemption of coupon for the HIV self-test, were given an Amazon online gift card for $25 in round 1 and $20 in rounds 2 and 3.

### Last HIV screening test

Of the 3197 eligible participants, 976 (30.5%) had never been tested for HIV prior to the HHTG, and another 1356 (42.4%) had been tested for HIV more than 6 months ago (see Table 2). It is important to note that the number of participants comprises the counts of unique participants as identified by e-mail address in each round of the HHTG, whereas some participants are counted twice or 3 times if they have participated in multiple rounds of the HHTG (see notes of Table 2 for details).

**TABLE 2:** Last HIV Test Among Eligible Participants, All 3 Rounds of NYS HHTG

	N^a^	0-3 Months Ago	4-6 Months Ago	7-12 Months Ago	12+ Months Ago	Never	No Answer/Not Sure
All	3197	8.4%	15.7%	16.8%	25.6%	30.5%	2.9%
Source of recruitment							
Grindr	1468	9.9%	18.7%	19.4%	22.1%	26.0%	3.9%
Facebook	979	7.8%	13.0%	14.4%	33.9%	28.0%	3.0%
Twitter	305	4.3%	11.8%	13.4%	21.3%	48.5%	0.7%
Instagram	112	11.6%	22.3%	14.3%	22.3%	28.6%	0.9%
Jack'd	98	3.1%	14.3%	19.4%	22.4%	37.8%	3.1%
Scruff	84	1.2%	9.5%	17.9%	23.8%	46.4%	1.2%
Black Gay Chat	48	0.0%	0.0%	12.5%	8.3%	79.2%	0.0%
Other	103	15.5%	17.5%	14.6%	26.2%	25.2%	1.0%
Race/ethnicity							
Asian or PI	133	13.5%	20.3%	14.3%	23.3%	26.3%	2.3%
Black	394	7.9%	14.7%	18.3%	19.8%	36.0%	3.3%
Hispanic	769	9.2%	17.9%	18.3%	19.6%	32.1%	2.7%
White	1730	8.3%	14.2%	16.4%	29.6%	28.6%	2.9%
Other/mixed	153	2.0%	20.9%	13.1%	30.1%	31.4%	2.6%
No answer	18	5.6%	11.1%	11.1%	11.1%	50.0%	11.1%
Age							
18-24 y	995	11.9%	16.5%	13.8%	13.2%	42.6%	2.1%
25-34 y	1260	7.5%	15.4%	18.3%	28.2%	27.4%	3.3%
35-44 y	428	5.8%	16.4%	15.4%	32.9%	26.6%	2.8%
45+ y	514	5.8%	14.4%	20.2%	37.5%	18.1%	3.9%
Gender^b^							
Cisgender men	3088	8.5%	15.3%	16.9%	26.0%	30.2%	2.9%
Cisgender women	0	...	...	...	...	...	...
TGNC/other-gender	109	3.7%	25.7%	13.8%	15.6%	38.5%	2.8%
Annual income^c^							
<$40 000	668	10.2%	21.0%	19.5%	22.8%	23.5%	3.1%
$40 000-$99 999	632	6.6%	13.4%	15.5%	23.1%	39.4%	1.9%
≥$100 000	86	10.5%	23.3%	19.8%	27.9%	12.8%	5.8%
Highest level of education^c^							
HS/GED or less^d^	245	7.3%	18.4%	21.2%	18.0%	29.8%	5.3%
Some college^e^	604	6.0%	14.6%	14.4%	22.4%	40.2%	2.5%
Bachelor's or higher	638	11.4%	20.8%	18.8%	25.4%	21.2%	2.4%

Abbreviations: HHTG, HIV Home Test Giveaway; NYS, New York State; PI, Pacific Islander; TGNC, transgender/gender nonconforming.

^a^The numbers of participants are the sums of the counts of the unique participants (by e-mail) in each round. Considering the participants who have participated in the HHTG more than once (using the same e-mail addresses), the total number of unique participants throughout the 3 rounds is 2963. Furthermore, because participants can enter the HHTG multiple times using different e-mail addresses, the actual number of unique participants may be smaller than 2963.

^b^Gender is measured by utilizing a 2-step method with sex at birth and current gender identity.

^c^Shows valid responses from the 1510 follow-up survey respondents.

^d^Includes “less than high school,” “some high school,” and “high school graduate or GED.”

^e^Includes “graduated from technical school” and “some college or an associate's degree.”

The percentage of individuals reporting having never been tested for HIV varied by social media recruitment source and by respondents' race/ethnicity, age, income, and education. More specifically, the percentage of individuals who had never been tested for HIV was 30.5% among the eligible participants, ranging from 26.0% among participants who were recruited from Grindr to 79.2% among the participants who were recruited from Black Gay Chat. Furthermore, the percentage who had not been tested for HIV in the past 12 months (including those who had never been tested) was 56.2% among the eligible participants, ranging from 48.2% among the Facebook users to 87.5% among the Black Gay Chat users. These findings should be interpreted with caution, because the sample size from Black Gay Chat was very small (only 48 participants), and also, important to note that half of the participants from Black Gay Chat identified as Hispanic/Latino, white, and other. Yet, black participants showed the highest rate of having never been tested for HIV (36.0%), significantly higher than the rates among Asians/Pacific Islanders (26.3%) or white participants (28.6%) at the .05 level.

Based on the information from the 1510 follow-up survey respondents, the association between having never been tested for HIV and income or education is implied to be inverse U-shaped. The percentage of having never been tested for HIV was the highest among the participants in the middle of the distributions of income (39.4%) and education (40.2%). Similarly, those who were in the middle of these distributions had the lowest rate of having been tested for HIV in the past 3 months.

Differences by sex at birth and gender were noted, but the sample size of some categories was too small for meaningful comparisons.

### HIV reactive test results

Of the 922 participants who reported having used the HIVST kit for themselves, 7 (0.8%) reported HIV reactive results. Of these, 6 reported that they had taken a confirmatory test: 5 self-reported they confirmed to be HIV positive and were linked to medical care; one was waiting for the confirmatory test results at the time of the follow-up survey. Table 3 shows their characteristics. Regarding their recent behaviors that put them at risk of HIV infection, 2 participants reported sex with partners who were known to be HIV positive and indicated using condoms and/or PrEP.

**TABLE 3:** Characteristics of 7 Participants With HIV Reactive Results, All 3 Rounds of NYS HHTG

	N
Source of recruitment	
Grindr	7
Facebook	0
Twitter	0
Instagram	0
Jack'd	0
Scruff	0
Black Gay Chat	0
Other	0
Race/Ethnicity	
Asian or PI	0
Black	2
Hispanic	2
White	2
Other/mixed	1
No answer	0
Age	
18-24 y	0
25-34 y	6
35-44 y	0
45+ y	1
Gender^a^	
Cisgender men	7
Cisgender women	0
TGNC/other-gender	0
Have health insurance	
Yes	7
No	0
Annual income	
<$20 000	2
$20 000-$39 999	4
$40 000-$59 999	1
≥$60 000	0
Highest level of education	
Some high school or less	2
High school graduate or GED	3
Some college or an associate's degree	1
4-y college degree or higher	1
Last HIV test	
0-6 mo ago	0
7-12 mo ago	3
12+ mo ago	1
Never	3
In the past 6 mo^b^	
Had condomless sex with men	4
Had sex with a partner with HIV infection	2
Diagnosed with STI^c^	1
Used narcotic drug	1
Used PrEP	2
Used PEP	0
Requested further assistance with^b^	
Additional HIV/STI prevention services	2
Other services	2
Saw a medical provider in the past 12 mo	
Yes	7
No	0

Abbreviations: HHTG, HIV Home Test Giveaway; NYS, New York State; PEP, postexposure prophylaxis; PI, Pacific Islander; PrEP, preexposure prophylaxis; STI, sexually transmitted infection; TGNC, transgender/gender nonconforming.

^a^Gender is measured by utilizing a 2-step method with sex at birth and current gender identity.

^b^Multiple categories may apply to the same participants.

^c^Chlamydial infection, gonorrhea, and/or syphilis.

### Participant follow-up requests

The NYS HHTG was enhanced after the first round to include a unique feature where participants were able to ask to be contacted via e-mail by the NYSDOH for additional services. In the first round of the NYS HHTG, participants were able to request for further assistance only if they reported the HIV reactive test result; none of the round 1 participants made such requests. Of the 1299 participants who completed the follow-up survey during the second and the third rounds of the HHTG, 761 (58.6%) requested assistance with various services from the NYSDOH. Individuals who requested assistance were contacted via e-mail, and 62 (8.1%) responded. Of those who responded, 23 (37.1%) did not request any services, 29 (46.8%) were referred to PrEP providers, and 10 (16.1%) were provided with other services, such as information on the HIV and sexually transmitted disease testing. None of those who reported HIV-positive results requested assistance from the NYSDOH (see Figure, Supplemental Digital Content 1, available at http://links.lww.com/JPHMP/A636, which shows these participants by their self-reported test results).

## Discussion and Conclusion

Overall, this online-based program design was effective at recruiting a large number of MSM within a relatively short period. The HHTG was highly accepted among the priority population, as 98% of participants who completed the follow-up survey and tested themselves reported that they would be likely to recommend the HHTG to a friend. Also, providing the HIVST kit at no cost was particularly crucial to encourage the priority population to be tested. Of those who reported having previously thought about purchasing a home test kit but did not do so, 62% reported the cost as a barrier. Also, our sample overrepresented lower-income earners; 6 of the 7 participants who tested HIV reactive earned less than $40 000 in the past year. In total, roughly 3000 eligible MSM were recruited, 63% of them redeemed the coupon for a free HIVST kit, and 29% reported having tested themselves. The HHTG was effective at ensuring individuals who wanted to test at home could do so and learn their test results in a timely fashion. Of close to 1000 individuals who reported having tested themselves for HIV, 0.8% reported HIV-positive (or preliminary positive) results.

Our sample showed high rates of having never been tested for HIV and having been tested for HIV longer than 12 months ago, compared with other studies.^10^,^14^,^17^,^18^,^20^,^25^,^29^ The percentage of the HHTG participants who had never been tested for HIV before was 34% among the total 6190 participants, 31% among the 3197 eligible participants, and 22% among the 922 participants who used the free HIVST kit for themselves. While the CDC stresses the benefits of more frequent screening (eg, every 3-6 months) for asymptomatic sexually active MSM,^30^ more than two-thirds of the eligible participants of our sample had not been tested for HIV for more than 6 months. Therefore, more HIV testing initiatives of similar programmatic design are encouraged.

In particular, it is noteworthy that the percentage of having never been tested for HIV greatly varied by the social media source where participants were recruited and was highest among the Black Gay Chat users 79.2%, followed by 48.5% among the Twitter users, 46.4% among the Scruff users, and 37.8% among the Jack'd users, whereas it was about 26% to 29% among the participants from Grindr, Facebook, and Instagram. Thus, HIV screening initiatives and programs may seek to better understand the user characteristics and tailor messaging and services to reach the users in smaller networks where a large number of priority population members may not adhere to CDC recommendations for HIV testing.

It is important to note that the NYS HHTG campaign ran simultaneously with the NYCDOHMH's Home Test Giveaway. This collaboration across the jurisdictional boundaries is one of the unique features of the HHTG, which is the first statewide HIVST initiative in the United States. Because many people residing outside of NYC visit NYC for play and/or work, simultaneously running home test giveaways in both NYC and NYS (outside of NYC) is key to the success of increasing HIV testing among NYS residents. The NYS HHTG data also suggest a great potential to extend collaboration with nearby states, because 23.9% of the total participants met all the eligibility criteria but were residing outside of NYS.

Another unique feature of the HHTG is that, since the second round of the HHTG, participants have been able to request assistance with additional services such as STI testing and PrEP referrals in the follow-up survey. This is a great way of addressing one of the concerns about HIV self-testing: the lack of opportunities for counseling, linkage to care, and STI testing.^4^ In the follow-up survey, the HHTG participants are able to request assistance with the following services: HIV confirmatory testing, PrEP, HIV/STI prevention services, and/or other support/supportive services. The HHTG participants who are confirmed HIV positive can further choose to request assistance with partner notification, HIV/AIDS medical care, and/or other services.

### Limitations

One of the major limitations of the follow-up survey was that many participants did not report their test results. While 7 participants reported their HIV reactive results, 17 participants did not report their rapid HIV test results, and 6 participants reported that their test results were indeterminate or not understandable. Some of these participants may have tested HIV reactive because some participants initially indicated an “HIV-positive” result and then came back to change their answer to “HIV negative” or “Prefer not to answer” after having completed some of the questions for those who tested HIV reactive.

Another limitation to the HHTG was its inability to identify duplicate participants. Because e-mail was the only identifiable information collected, participants were able to enter the system multiple times using different e-mail addresses and order more than 1 HIVST kit (ie, some may want another test kit for a later use or to give to a partner and/or friend or family member who may be at elevated risk of HIV/AIDS). Consequently, the actual count of the unique participants might be smaller than what was reported in this article.

Still another limitation is related to the fact that this article is based on participants' self-reported data. Accordingly, their survey responses may be subject to social desirability bias, recall errors, misunderstanding of questions, and confidentiality concerns.

**Implications for Policy & Practice**

- Social media and dating mobile applications provide a venue for reaching gay and bisexual men and MSM within a relatively brief period, some of whom may not have been reached otherwise. Thus, HIV screening initiatives and programs should consider these channels for distributing rapid HIVST kits.
- While there are popular dating mobile applications (such as Grindr) that attract many MSM, targeting smaller networks (eg, Black Gay Chat) may also be effective if their members do not test for HIV frequently.
- Many participants comment that HIVST kits are too expensive to use as often as needed. Distribution of free HIVST kits is key to expanding HIV screening among the MSM population.
- Campaign messages and images have been tailored to the various groups of our priority populations, including TG/GNC individuals, who are hard to reach and engage in HIV prevention programming. Our program model shows promise that with additional consumer input and strategic advertisement placements, the HHTG has the potential to reach TG/GNC individuals in NYS.
- The HHTG has positive implications for public health as an important tool for improving sexual health, especially for the most stigmatized individuals who are not willing or able to access traditional services.

## Supplementary Material

SUPPLEMENTARY MATERIAL
