# Supplementary material for: Combating Stigma Through HIV Self-Testing: New York State's HIV Home Test Giveaway Program for Sexual Minorities
Source: J Public Health Manag Pract. 2020 Feb 3;28(2):174–83. doi: 10.1097/PHH.0000000000001138 (PMC8781215; doi:10.1097/PHH.0000000000001138)
Supplement: SUPPLEMENTARY MATERIAL [file jpump-28-0174-s001.docx]

**Supplement Digital Content 1** Participant Follow-up Requests for Rounds Two and Three of the NYS HHTG
